# Deprescribing Following Access to Lifestyle Treatment: A Retrospective Chart Review of Primary Care Outcomes in Patients with Type 2 Diabetes

**DOI:** 10.3390/jcm15072561

**Published:** 2026-03-27

**Authors:** Yoav Jacob, Kara L. Staffier, Samveda Menon, Puja B. Gandhi, Joeita F. MacField, Gia Merlo, Stefanie M. Meyer, Shivani S. Patel, Caroline Rhéaume, Madeline Watson, David Donohue, Wayne S. Dysinger, Micaela C. Karlsen

**Affiliations:** 1 Albert Einstein College of Medicine, Bronx, NY 10461, USA; yoav.jacob@einsteinmed.edu; 2 Department of Research, American College of Lifestyle Medicine, Chesterfield, MO 63017, USA; 3 University of Connecticut, Storrs, CT 06269, USA; 4 McGovern Medical School, UT Health, Houston, TX 77030, USA; puja.gandhi@uth.tmc.edu; 5 College of Human Medicine, Michigan State University, Grand Rapids, MI 49503, USA; 6 Department of Psychiatry, NYU Grossman School of Medicine, New York, NY 10016, USA; giamerlomd@gmail.com; 7 Department of Nutrition, Dietetics, and Exercise Science, College of Health Professions, Concordia College, Moorhead, MN 56562, USA; smmeyer@cord.edu; 8 Rowan Virtua School of Osteopathic Medicine, Stratford, NJ 08084, USA; 9 Department of Family Medicine and Emergency Medicine, Faculty of Medicine, Université Laval, Quebec City, QC G1V 0A6, Canada; caroline.rheaume@fmed.ulaval.ca; 10 College of Osteopathic Medicine of the Pacific, Western University of Health Sciences, Pomona, CA 91766, USA; 11 Progressive Health of Delaware, Wilmington, DE 19810, USA; 12 Blue Zones Health, Riverside, CA 92506, USA; 13 Departments of Applied Nutrition and Global Public Health, University of New England, Biddeford, ME 04005, USA

**Keywords:** lifestyle medicine, deprescribing, primary care, medication de-escalation, type 2 diabetes

## Abstract

**Background**: Among individuals with type 2 diabetes (T2D), lifestyle improvements can restore glycemic control, yet few studies have examined deprescribing in settings where it was necessitated by improvements in health. This study aimed to (1) identify instances of medication deprescribing among adults with T2D in a primary care setting where patients had access to lifestyle medicine (LM), (2) document lifestyle changes among deprescribed patients, (3) assess changes in body mass index (BMI), glucose, and hemoglobin A1c (HbA1c) following deprescribing, and (4) assess the safety of deprescribing in the context of LM-informed care by identifying adverse events. **Methods**: A retrospective review of electronic health records (EHR) was conducted among 650 adults with a diagnosis of T2D per ICD-10 code at two primary care practices. To be included in the study, individuals had to be seen at least two times during the study period, from 2014 to 2023. Using a previously developed deprescribing framework, records were reviewed to identify deprescribing events. Among patients who were identified as deprescribed, BMI, glucose, and HbA1c, were extracted from the EHR, and age-, sex-, and time-adjusted differences in least squares means were calculated. Mentions of lifestyle change in provider notes in the EHR were also extracted pre- vs. post-deprescribing. **Results**: Forty-one deprescribing events were confirmed, totaling 6.3% of the study population. The most common medication changes included metformin dose reduction 34%, metformin discontinuation 19.5%, and insulin dose reduction 19.5%. Among patients with follow-up data, mean BMI decreased by 2.25 kg/m^2^, *p* = 0.0003. Mean decreases of 25% in glucose and 13% in HbA1c were also observed, *p* < 0.0003 and *p* < 0.0013, respectively. Lifestyle modifications were specifically cited in 51% of records among deprescribed patients, most frequently related to diet and exercise. No serious adverse events were identified in patients who were deprescribed. **Conclusions**: In a primary care setting where patients had access to lifestyle medicine, a subset of adults with T2D experienced meaningful health improvements and were able to reduce glucose-lowering medications without any serious adverse events noted in the EHR.

## 1. Introduction

Deprescribing, the process of tapering, reducing, or discontinuing medications that may be inappropriate or unnecessary, is an increasingly recognized strategy to address polypharmacy and optimize patient outcomes [1]. This process is increasingly relevant in type 2 diabetes (T2D), a chronic metabolic disorder affecting over 35 million Americans [2]. While glucose-lowering medications remain central to T2D management, reliance on pharmacotherapy does not treat the root cause of the disease and can lead to side effects, treatment burden, and increased healthcare costs [3,4,5,6,7,8].

Lifestyle medicine (LM) is an evidence-based clinical discipline that uses therapeutic lifestyle interventions, including a whole-food, plant-predominant diet, regular physical activity, restorative sleep, stress management, risky substance avoidance, and positive connectedness as the primary modality for preventing, treating, and even reversing chronic disease [9]. LM care is rooted in a set of 89 core competencies outlining the knowledge and skills required to practice [10]. LM is versatile in that it can be delivered across a variety of settings, at varying intensities, and using multiple modalities [11]. The most intensive delivery is in the form of intensive, therapeutic lifestyle change (ITLC) delivered by lifestyle medicine intensivists [12]; however, LM can also be integrated at varying levels of intensity in settings such as primary care [13]. Health improvements resulting from LM can trigger medications to become unnecessary or require dose reductions as patients achieve improved disease markers, indicating glycemic control [14]. As such, deprescribing has emerged as a clinically meaningful, patient-centered strategy to reduce medication burden, minimize adverse effects, and practice patient-centered, evidence-based care [15,16,17,18,19].

Despite advancements in care, significant gaps remain in measuring outcomes following lifestyle interventions, particularly in capturing when such improvements justify pharmacologic dose reductions or discontinuation. Most existing frameworks are primarily designed to address the risks of polypharmacy in older adults and do not account for lifestyle-driven clinical improvements that reduce medication needs. Furthermore, most previous deprescribing investigations have tended to focus on a single drug, drug class, or niche patient population, often within highly specialized teams [14,18,20]. One small study, for example, explored deprescribing following intensive interventions such as bariatric surgery [21]. However, while such findings underscore the potential for clinical improvement to warrant medication reduction, the surgical context is not broadly applicable. Broader medication reviews, when conducted, typically require multidisciplinary input and are not yet routine in general practice. While clinicians frequently describe personal decision-making processes during medication reviews, few reported using formal protocols or external deprescribing tools, even when such tools are available and recommended. However, empirical evaluations of medication changes in which medication reduction follows intentional or incidental improvements in health behaviors are critically needed [22].

For many patients, the possibility of reducing or eliminating medications can serve as a powerful motivator for engaging in lifestyle change [23]. However, this outcome has rarely been captured or studied systematically, despite its clinical and psychosocial significance. To address this gap, we previously developed a framework to identify cases of deprescribing to facilitate future research on the topic, particularly in relation to health outcomes [24]. This study takes the next step, utilizing the framework to characterize types of deprescribing and then examining health outcomes among patients identified as deprescribed.

The objectives of this study are to (1) use a previously developed deprescribing framework to identify types of medication deprescribing among adults with T2D in a primary care setting where patients had access to LM, (2) document lifestyle changes among deprescribed patients, (3) assess changes in BMI, glucose, and HbA1c following deprescribing, and (4) assess the safety of deprescribing in the context of LM-informed care by identifying adverse events.

## 2. Methods

This study applies a previously developed framework for identifying and classifying instances of deprescribing of glucose-lowering medications [24]. In brief, two primary care practices that offer LM as part of routine clinical care and share an electronic health record (EHR) platform participated by providing records from 650 patients (ages 18–89 years). Included patients were those with a diagnosis of T2D defined by an ICD-10 code and had at least two clinical encounters in the EHR during the analysis period, which ranged from 2014, the time of the inception of the EHR, through 2023, when this study began. The sample size comprises all data available during this time period that meet the inclusion criteria. No further exclusions were made. A small number of patients were missing documented BMI, glucose, and/or HbA1c in the EHR for a relevant time point(s), and analyses of clinical outcomes are presented using available data as specified in Table 1.

An affiliated programmer prepared a deidentified EHR data export of all patient encounters for this cohort. A multidisciplinary review team consisting of physicians, medical students, researchers, and a pharmacist reviewed the records to understand medication changes. At least two team members independently reviewed each patient’s records, and the study team met regularly to discuss findings and resolve discrepancies. Possible instances of deprescribing, based on medication prescription changes and physician notes, were identified, and a framework for classifying the type of deprescribing was iteratively developed based on consensus of the team, with multiple revisions during the record review.

A team member (SM), who was also an employee of one of the primary care practices, reviewed the EHR for information related to deprescribing, including medications prescribed, dates, doses, and provider notes for patients who were initially identified as potentially deprescribed. At the same time, these patient records were also reviewed for adverse events related to medication changes, and lifestyle changes noted in the EHR were documented. After reviewing all possible patient records, confirming whether patients had been deprescribed, and determining how to classify them based on the framework, two other team members (KS and MK) met for a discussion of cases that were unclear, and made a consensus-based decision on how to classify them.

For this same cohort of patients confirmed to have been deprescribed, BMI, glucose, and HbA1c, at the first and last (i.e., most recent follow-up) timepoints during the study period were extracted from the EHR into a spreadsheet. Least squares means were calculated using a mixed model, adjusted for age, sex, and time (days between pre- and post-deprescribing timepoints), with *p* < 0.05 considered statistically significant. Glucose and HbA1c were log-transformed and exponentiated back to the original scale for presentation. This study was reviewed by the University of New England Institutional Review Board.

## 3. Results

**Figure 1:**
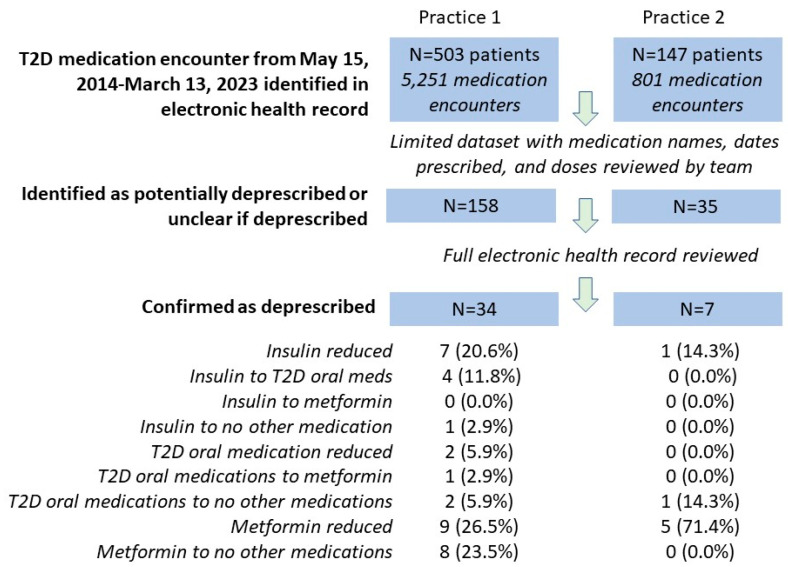
Summary of deprescribing events.

A total of 650 patients with type 2 diabetes (503 from practice 1, 147 from practice 2) were identified, representing 6052 medication encounters between May 15, 2014, and March 13, 2023 (see Figure 1: Summary of Deprescribing Events). From these encounters, 193 were initially flagged as potentially deprescribing or unclear (158 from practice 1 and 35 from practice 2). Following EHR review, 41 cases (6.3%) were confirmed as deprescribing (34 from practice 1 and 7 from practice 2) based on the deprescribing framework [24]. Of the 41 participants confirmed as deprescribed, 18 (44%) were male, and the mean (SD) age was 67.4 (11.3) years, range 42–91 years.

Among deprescribing cases in practice 1, the most frequent change was metformin dose reduction (26.5%), stopping metformin (23.5%), and insulin dose reduction (20.6%). Other changes included transitions from insulin to oral agents (11.8%), reduction in other oral diabetes medications (5.9%), discontinuation of oral agents without replacement (5.9%), and transitions from insulin or oral medications to metformin (2.9%). In practice 2, the most common change was metformin dose reduction (71.4%), followed by insulin dose reduction (14.3%) and discontinuation of oral medications without replacement (14.3%). Overall, the most common medication changes included metformin dose reduction (34%), metformin discontinuation (19.5%), and insulin dose reduction (19.5%).

Clinical outcomes associated with deprescribing were evaluated among patients with available follow-up data (see Table 1: Comparisons of Outcome Pre- vs. Post-Deprescribing). The mean follow-up time was 47 months (3.9 years). Across 39 patients with available BMI records, mean BMI was 2.25 kg/m^2^ lower after being deprescribed, when controlled for age, sex, and days difference between the pre- and post-deprescribing timepoints (*p* = 0.0003) A mean 25% decrease in glucose (*p* = 0.0003) and 13% decrease in HbA1c (*p* = 0.0013) were observed from pre- to post-deprescribing, after controlling for age, sex, and days difference between timepoints.

**Table 1:** Comparisons of outcomes pre- vs. post-deprescribing ^a^.

	N	Differences in LS Means (95% CI)	*p*-Value
**BMI (kg/m^2^)**	39	−2.25 (−3.39, −1.11)	0.0003
**Glucose (mg/dL)**	36	0.75 (0.65, 0.87) ^b^	0.0003
**HbA1c (%)**	33	0.86 (0.79, 0.94) ^b^	0.0013

^a^ Age-, sex-, and time-adjusted; ^b^ glucose and HbA1c were log-transformed; LS means and 95% CI are exponentiated back to their original scale for presentation.

Three adverse events were noted during chart review. These included increases in HbA1c and glucose in one patient, concurrent weight gain and HbA1c increase in another patient, and episodes of hypoglycemia in a patient taking tizanidine (Zanaflex), started by a separate pain specialist. Tizanidine is not known to directly cause hypoglycemia or to have clinically significant interactions with common type 2 diabetes medications. However, indirect effects (e.g., sedation, reduced oral intake, or missed meals) may contribute to hypoglycemia, particularly in patients using insulin or insulin secretagogues. Through the chart review, these events were determined to be unrelated to the effects of deprescribing within the lifestyle medicine practices’ clinical context.

In the EHR, lifestyle changes were explicitly documented in 21 of the 41 patient records (51%) with confirmed deprescribing. One record referenced lifestyle change in general terms without detail, 11 specifically documented dietary change guidance, and 9 documented discussions with patients regarding both diet and exercise. The practice administrative physician confirmed that notetaking was sometimes inconsistent among providers, and that patients who engaged in lifestyle changes may not have had these changes consistently recorded in the EHR.

## 4. Discussion

We examined medication deprescribing among adults with T2D in two primary care practices. Of 650 patients reviewed, 41 (6.3%) were confirmed to have undergone deprescribing of diabetes medications following clinical improvements, including weight loss and reductions in HbA1c levels. This percentage, while small, is clinically meaningful, as these findings establish that deprescribing is not only possible but can emerge as a direct and measurable clinical outcome in a primary care practice where LM is available to patients, thus underscoring lifestyle interventions’ relevance for patient safety and care optimization [25]. For these patients, lifestyle changes were sufficient to produce measurable changes in glycemic control that required medication adjustment, even when patients were not necessarily seeking lifestyle-focused treatment. These findings are relevant amid prior studies suggesting that patients with T2D are open to deprescribing, in some cases, when paired with perceived improvements in health and well-being [26,27,28].

Our findings align with previous reports, as well as a recent clinical practice guideline, highlighting the central role of lifestyle medicine in T2D treatment [29,30,31] and extend the literature by documenting this relationship in a real-world, primary care setting where LM is available to patients. Most notably, the patients in this study who experienced deprescribing had not necessarily sought LM-informed care, underscoring the potential for routine LM integration in primary care to shift treatment trajectories, even in patients without preexisting interest in non-pharmacologic approaches. Our results challenge assumptions that lifestyle interventions are impractical or ineffective in routine care and suggest that a meaningful proportion of patients—over 6% in this sample cohort—are both willing and able to achieve clinically significant improvements when offered lifestyle treatment as part of routine primary care. If these results were scaled nationally to the millions of adults with T2D, even a 6% deprescribing rate represents a substantial public health impact. Reductions in medication use can lower treatment burden for patients and could generate meaningful cost savings for payers [32].

Among those who were deprescribed, the mean age was 67.4 years, with most being older adults, a population most frequently cited in traditional deprescribing studies due to polypharmacy risks [1,16,17,33]. However, unlike prior work, where deprescribing was often driven by adverse events or end-of-life considerations [15,22,34], our analysis highlights a proactive model centered on clinical improvement. For example, patients experienced a statistically significant mean decrease in BMI of 2.25 kg/m^2^, as well as a mean 25% decrease in glucose and a mean 13% decrease in HbA1c. Clinician notes frequently cited lifestyle changes as contributing factors, with 51% of patients having documentation of lifestyle guidance or behavioral change, most related to diet and exercise. This proportion likely underestimates the true prevalence of lifestyle involvement, reflecting documentation practices in these LM practices rather than an absence of lifestyle counseling. This is particularly notable given that current deprescribing frameworks do not specifically consider lifestyle change as a primary trigger for medication reduction [14,35].

The most frequently deprescribed medication was metformin, either reduced (34%) or discontinued altogether (19.5%), followed by reductions in insulin or oral hypoglycemics. The frequent reductions in metformin are consistent with recommendations in the American Diabetes Association’s Standards of Care Guidelines to use metformin as first-line treatment or in combination with other T2D medications [36]. In several cases, patients were successfully transitioned from multiple agents to metformin alone or to no medications at all.

Concerns around patient safety, particularly the risks associated with withdrawing medications, remain insufficiently studied, especially in populations where chronic conditions may effectively be treated through intensive lifestyle change regimens [34]. Deprescribing should consider the full therapeutic profile of medications, including cardioprotective benefits, and that glycemic improvement alone may not be sufficient justification for discontinuation in all patients. Some literature overviews patient perspectives, including their interest in, comfort with, and perceived benefits of deprescribing, particularly when lifestyle interventions have improved disease control [33]. Patients, especially those with cardiovascular disease or diabetes, have been found to be open to deprescribing, but this interest is frequently contingent on whether the provider initiates the conversation [26,27]. In our study, there was only 1 instance of hypoglycemia, and the effects were mild and resolved without trouble. It is the position of the authors that the benefits of lifestyle changes far outweigh the potential risk of temporary hypoglycemic episodes that may occur before medications have been sufficiently de-escalated.

Deprescribing has been linked to improved quality of life and enhanced physical and emotional well-being, particularly among older adults [37,38]. Yet, such examples remain the exception rather than the norm. Despite possible patient receptiveness, deprescribing discussions are not consistently incorporated into routine clinical encounters. Medication-focused visits may be viewed as redundant or unnecessary, especially if patients believe their existing care already addresses their medication needs [26]. Determining the best method to initiate deprescribing found empowering patients with confidence to ask, as potentially beneficial, but this work has had a limited clinical scope [28,39]. Efforts to develop feedback reports or similar digital tools that provide deprescribing recommendations have had a small preliminary impact [18,40]. As a result, opportunities to reassess medication necessity, particularly in the context of lifestyle-induced clinical improvements, may go missed.

Our study offers several strengths, including a large retrospective cohort (n = 650 patients, 6052 medication encounters) drawn from two established primary care practices where LM is available to patients, with consistent use of EHRs, allowing for standardized data review. Our multi-disciplinary team of clinicians, researchers, medical students, and a pharmacist provided the skills and knowledge needed to interpret and categorize patient records. The application of a structured deprescribing framework [24] helped ensure clarity and consistency during data extraction.

However, our findings should be interpreted with several limitations in mind. Although patients were not specifically recruited based on interest in LM, they may represent a somewhat self-selecting group more open to lifestyle interventions than the general population. While the practices in this study provided detailed records, such records may not be consistently available in other settings, potentially limiting reproducibility. Although the retrospective cohort used to assess the number of patients deprescribed vs. not deprescribed was large, the number of patients for which health outcomes were analyzed is relatively small (n = 41). While 51% of the deprescribed patients had mention of lifestyle in their EHR, we cannot conclusively determine the impetus for deprescribing in the other 49%. It is relevant, however, that in both of these practices, lifestyle intervention is offered to all patients if they are interested, and it would be uncommon to see improvements in health metrics such as HbA1c without increased medication use if no lifestyle changes were involved. Although our retrospective design enabled us to examine real-world practice, it limits our ability to determine causality and to fully capture patients’ perspectives, particularly regarding their experiences with deprescribing, their role in decision-making, and the perceived benefits or risks of medication reduction. Lastly, EHR access was limited to data fields related to deprescribing; thus, we did not have access to all demographic data or information on the duration of T2D in the patients assessed.

Future research could build on a new clinical practice guideline that supports proactive deprescribing in LM-informed care [31]. The adoption of deprescribing protocols that respond to lifestyle-induced improvements (e.g., glycemic control, weight loss) could further enhance patient-centered, evidence-based chronic disease care. As deprescribing gains recognition as a clinical and research priority, it will be important to solidify its scope beyond risk mitigation, to also include improvement-based approaches rooted in lifestyle change. Studies are needed to prospectively test structured deprescribing protocols following lifestyle change, further define best practices, inform clinical protocols, and support the integration of deprescribing as a measurable endpoint in both LM-focused and broader care settings. Ultimately, integrating lifestyle-driven deprescribing into clinical frameworks may strengthen efforts toward remission-based models of chronic disease management.

## 5. Conclusions

This study identified multiple instances of glucose-lowering medication reduction among adults with T2D in a primary care setting where patients had access to LM. Documented lifestyle changes, most frequently diet and/or physical activity changes, were specifically noted among 51% of deprescribed patients. Our findings suggest that deprescribing can be safely and appropriately implemented in routine care and may serve as a valued marker of success for patients and clinicians.

## Data Availability

In accordance with the IRB approval and to ensure confidentiality of patients, data is not available to members outside of the study team.

## Abbreviations

The following abbreviations are used in this manuscript:

T2D	Type 2 diabetes
LM	Lifestyle medicine
EHR	Electronic health record
ACLM	American College of Lifestyle Medicine
